# Evaluation of sleep disorder in orthopedic trauma patients: a retrospective analysis of 1129 cases

**DOI:** 10.1186/s13018-021-02487-2

**Published:** 2021-05-29

**Authors:** Hai Yang, Yi-jia Liu, Jia-lu Ye, Li-hong Zhao, Ling-li Li, Xiao-ling Hou

**Affiliations:** 1 Department of Orthopedics, West China Hospital of Sichuan University, Chengdu, Sichuan People’s Republic of China; 2 School of Nursing, West China Hospital of Sichuan University, Chengdu, Sichuan People’s Republic of China; 3 Mental Health Center, West China Hospital of Sichuan University, Chengdu, Sichuan People’s Republic of China

**Keywords:** Orthopedic trauma, Sleep disorder, Sleep, Pittsburgh sleep quality index

## Abstract

**Background:**

In the trauma center wards, it is not unusual for patients to have sleep disorders, especially patients with an acute injury. Meanwhile, there is substantial evidence that sleep disorder is a predictor of depression and is an important feature of posttraumatic stress disorder.

**Methods:**

All orthopedic trauma patients confined in a trauma ward in West China Hospital of Sichuan University between April 2018 and July 2019 were included in this retrospective study. Patients with mental impairment or craniocerebral injuries were excluded from the study. Basic demographic data and the Injury Severity Score (ISS) classification based on medical records were collected. The Pittsburgh sleep quality index (PSQI) was used to evaluate sleep quality, the visual analog scale (VAS) was used to evaluate physical pain, and the Barthel Index (BI) was used to evaluate activities of daily living (ADL). Univariate linear regression analysis and multivariate linear regression analysis were used to identify independently related factors.

**Results:**

The average PSQI score was 6.3 (± 4.0). A total of 581 (51.4%) patients had a PSQI score > 5, indicating the presence of sleep disorders. The PSQI score was > 10 in 174 (15.4%) patients. Univariate statistical analysis showed that age, sex, education, ADL, and ISS classification were associated with increased PSQI scores. Marital status and pain were not associated with increased PSQI scores. When we used multivariate analysis to control for confounding variables, sex, ADL, and ISS classification remained independently associated with PSQI (P = 0.002, < 0.000, and 0.002, respectively).

**Conclusions:**

In our study, sleep disorders were common (51.4% with PSQI > 5) and serious (15.4% with PSQI > 10) in patients with traumatic orthopedic injury. The following factors were closely associated with sleep disorders: sex, ADL, and ISS classification. Moreover, age and educational attainment have an independent impact on sleep quality. Unexpectedly, the VAS score for pain was not independently associated with the seriousness of sleep quality, which may be related to preemptive and multimodal analgesia. Further studies are required to clarify this ambiguity.

## Background

Sleep disorders are highly prevalent after physically traumatic events and closely related to posttraumatic stress disorder (PTSD) and depression [1, 2]. Patients who suffer from trauma often require treatment for insomnia, nighttime arousals, and nightmares [3, 4]. In our experience, we observed that most orthopedic trauma patients cannot fall or stay asleep easily and quickly. Difficulty falling or staying asleep is an important component of PTSD because of repetitive nightmares caused by hyperarousal and intrusive processes [5].

Sleep disorders have serious impacts on general health, quality of life, and physical functioning [6, 7], resulting in physical disability [7]. Therefore, it can exacerbate the physical and functional limitations of orthopedic trauma patients. A cohort study indicated a strong correlation between physical disability and long-term psychological stress that combined poor sleep quality [8]. As treatment for sleep disorders in orthopedic trauma patients may not be beyond the scope of surgical practice, this serious traumatic complication cannot be ignored. We have a duty to determine the prevalence of sleep disorders in this patient population.

Many articles and reviews have shown that sleep disorders are caused by trauma, especially in patients with PTSD [1, 3, 4, 9–11]. We can also find literature related to the association between orthopedic trauma and depression [12]. Although sleep disorders may be the core feature of depression or PTSD [13], few studies evaluated sleep quality in orthopedic trauma patients, and only Swann et al. studied the factors and severity of sleep disorders in orthopedic trauma patients [14]. Moreover, there are no related studies from China on patients suffering from acute orthopedic trauma. This study aimed to evaluate the prevalence of sleep disorders in patients in the ward of a trauma center who suffered from an acute orthopedic injury. We also tried to identify factors associated with sleep disorders, such as patient features, injury severity, and physical pain.

## Materials and methods

### Subjects

Following approval by our institutional review board, we conducted a cross-sectional and retrospective study of patients with orthopedic trauma in the ward of the trauma center of West China Hospital of Sichuan University. All patients who were admitted to the emergency department with acute physical injury, including acute closed fracture, acute open fracture, and skin soft-tissue defect with or without multiple trauma, between April 2018 and July 2019 were included. To ensure the accuracy of data, the exclusion criteria included the following: pediatric patients (0–11 years old), patients with mental impairment, craniocerebral injury, and other conditions that hindered them from completing the evaluation, and those whose electronic medical records were missing.

All eligible patients were evaluated for Pittsburgh sleep quality index (PSQI), physical pain on the visual analog scale (VAS), Barthel Index (BI), and Injury Severity Score (ISS) classification by nurses within 3 days of admission, and all related and demographic data of these patients were registered in the medical records we collected.

### Main outcome measurements

The PSQI questionnaire is an effective method for assessing a patient’s sleep quality [15]. Nineteen items generate seven scores: sleep latency, sleep duration, habitual sleep efficiency, subjective sleep quality, use of sleeping medication, sleep disturbances, and daytime dysfunction. Then, we summed all parts of the scores; the total score was 21. For this study, we used PSQI > 5 as an indicator of sleep disorder, and PSQI > score 10 as severe sleep disorder. We expected the impact of sleep disorders and physical pain levels. We used the VAS to assess physical pain in patients with acute orthopedic trauma [16, 17]. The VAS was categorized as follows: no pain (0), mild pain (1–3), moderate pain (4–6), and severe pain (7–10).

The BI was used to assess the activities of daily living (ADL) of patients [18]. We used the 10-item scale, which included bathing, grooming, dressing, feeding, toilet use, transfers, mobility, bowels and bladder movement, and climbing the stairs. A total score of 0 indicated total dependence and a score of 100 indicated complete independence. In our study, we used the BI categorized as follows: severe dependence (0–40), moderate dependence (41–60), and mild dependence (61–99). Finally, we used the ISS and Abbreviated Injury Scale (AIS)-2015 to evaluate the traumatic level of patients [19, 20]. The precise ISS scores of patients could not be extracted from medical records, but we could obtain the ISS classification, which was registered in the nursing assessment sheet. The ISS was categorized as follows: severe injury (≥ 25), moderate injury (16–24), and mild injury (< 16).

### Statistical analysis

Statistical analysis was performed using SPSS v.22 statistical software (IBM, Armonk, NY, USA). Demographic data and data on patient features were obtained, including age, sex, marital status, and education. We divided the educational attainment of patients into 10 grades as follows: below primary school, primary school, junior high school, technical secondary school, senior high school, junior college, bachelor’s degree, master’s degree, doctorate, and other. All categorical variables were set as dummy coding and substituted into the linear regression calculation. Mean and standard deviation (SD) or median and interquartile ranges were reported. Univariate linear regression was used to determine the potentially relevant variables for the sleep disorder. Relevant variables verified by the univariate analysis were included in a multivariate linear regression analysis to select for confounding variables and to isolate independent variables of sleep disorders.

## Results

Medical records of 1129 patients were included in the analysis, of which 680 (60.2%) were men and 449 (39.8%) were women. The average age was 50.4 ± 19.6 years, the male average age was 46.03 ± 17.6 years, and the female average age was 57.03 ± 20.5 years. A total of 886 (78.5%) patients were married, 152 (13.4%) were unmarried, and 91 (8.1%) were divorced or widowed. The total educational attainment was not high. Seventy-four (6.6%) patients did not graduate from primary school, 281 (24.9%) graduated from primary school, 331 (29.3%) graduated from junior high school, 68 (6.0%) graduated from technical secondary school, 130 (11.5%) graduated from senior high school, 101 (8.9%) from junior college, 115 (10.2%) have a bachelor’s degree, 13 (1.2%) have a master’s degree, and 2 (0.2%) have a doctorate. More than half of the patients (636, 56.3%) had pure closed fractures, 182 (16.1%) had open fractures, 140 (12.4%) had multiple traumas, 59 (5.2%) had severely damaged trauma and needed amputation, 79 (7.0%) had pure soft tissue defects, and 33 (2.9%) had a pure dislocation.

**Fig. 1:**
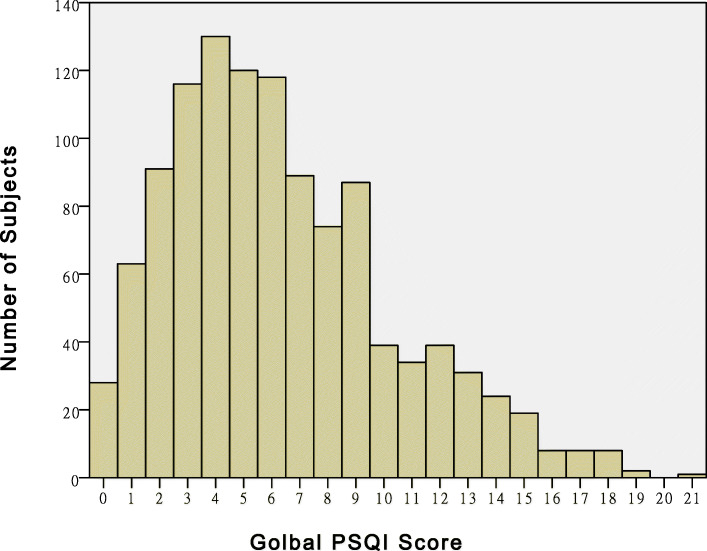
PSQI score distribution

We also analyzed the VAS, BI, PSQI, and ISS classification in the medical records. A total of 923 (81.8%) patients were mildly injured, 154 (13.6%) were moderately injured, and 52 (4.6%) were severely injured. The average BI score was 36.7±21.5. Most patients (71.2%) were severely dependent, 14.1% were moderately dependent, and 13.9% were mildly dependent. The average VAS score was 2.3 ± 1.0. Most patients (89.0%) experienced mild pain, and 3.8% felt moderate pain. The average PSQI score was 6.3 ± 4.0. The distribution trends of the PSQI scores are shown in Fig. 1. Sleep disorders (PSQI > 5) were noted in 581 (51.5%) patients. The PSQI score was > 10 in 173 patients (15.4%). A summary of patient demographics and other scores is presented in Table 1.

**Table 1:** Demographic data and patient-reported outcome measures

Variables	
**Age (years)**	50.4 ± 19.6
**Sex**	
Male	680 (60.2%)
Female	449 (39.8%)
**Marital status**	
Married	886 (78.5%)
Unmarried	152 (14.3%)
Divorced or widowed	81 (7.2%)
**Types of injury**	
Closed fracture	636 (56.3%)
Open fracture	182 (16.1%)
Severely damaged trauma	59 (5.2%)
Multiple trauma	140 (12.4%)
Skin soft tissue defect	79 (7.0%)
Joint dislocation	33 (2.9%)
**Educational level**	
Below primary school	74 (6.6%)
Primary school	281 (24.9%)
Junior high school	331 (29.3%)
Technical secondary school	68 (6.0%)
Senior high school	130 (11.5%)
Junior college	101 (8.9%)
Bachelor	115 (10.2%)
Master	13 (1.2%)
Doctor	2 (0.2%)
**VAS**	2.3 ± 1.0
≤ 3	1085 (96.1%)
> 3	47(3.9%)
**BI (ADL)**	36.7 ± 21.5
0-40 (severe dependence)	804 (71.2%)
41-60 (moderate dependence)	160 (14.1%)
61-100 (mild dependence or no dependence)	165 (14.7%)
**ISS classification**	
Mild injury	923 (81.8%)
Moderate injury	154 (13.6%)
Severe injury	52 (4.6%)
**PSQI**	6.3 ± 4.0
> 5	581 (51.5%)
> 10	173 (15.4%)
**Total**	1129 (100%)

Note: If joint dislocation and fracture occurred simultaneously, it was considered as a fracture. All patients with severe trauma were amputated. Patients with multiple traumas were not amputated. Patients with skin soft tissue defects did not combine with any fracture or joint dislocation

Table 2 shows the results of the univariate linear regression analysis of PSQI. Age, sex, marital status, educational attainment, DRG, pain, ADL, and ISS classification were associated with PSQI in univariate analysis. These variables were then evaluated using multivariate linear regression analysis. However, only sex, ADL, and ISS classification were independent predictors of PSQI. Age and education were not independently associated with PSQI in multivariate analysis, indicating that the significant variables in univariate analysis were influenced by confounding variables. Unexpectedly, VAS was not vitally associated with PSQI in univariate analysis. The detailed data of the multivariate analysis are shown in Table 3.

**Table 2:** Univariate linear regression of PSQI

Variables	B	T	P
**Age**	0.014	2.397	0.017
**Sex**	−0.764	−3.183	0.001
**Marital status**	0.143	0.138	0.473
**Educational level**	−0.171	−2.739	0.006
**Pain**	−0.013	−0.116	0.908
**ADL**	−0.038	−7.019	0.000
**ISS classification**	1.066	4.723	0.000

**Table 3:** Multivariate linear regression of PSQI

Variables	B	T	P
**Age**	0.011	1.644	0.100
**Sex**	−0.766	−3.137	0.002
**Education**	−0.027	−0.404	0.686
**ADL**	−0.032	−5.431	0.000
**ISS classification**	0.741	3.108	0.002

## Discussion

In our study, sleep disorders were common (51.4% with PSQI > 5) and serious (15.4% with PSQI > 10) in patients with acute traumatic orthopedic injury. Muscatelli revealed the incidence of depression and PTSD in patients with acute orthopedic trauma through a systematic review that included 7109 subjects in the analysis. They confirmed that in patients with acute orthopedic trauma, the weighted combined prevalence of depression and PTSD was 16.8% [12]. By comparing the results of our study with the data of that study, it can be gathered that the proportion of patients with severe sleep disorders is similar to the incidence of PTSD and depression. Some studies have shown that sleep disorders are closely related to PTSD and depression [1, 2], we believe that severe sleep disorders are associated with PTSD and depression. At the same time, PSQI scores > 10 could be regarded as an important indicator for predicting PTSD in patients with acute orthopedic trauma.

Interestingly, we found that the VAS score for physical pain was not correlated with sleep quality in our research results. The average VAS score was 2.3 ± 1.0, which is a low-level score. This is inconsistent with our previous experience, and the literature also pointed out that pain is closely associated with poor sleep quality [21, 22]. To determine what lead to this result, we checked the relevant medical records of inpatient and emergency departments. We found that the treatment of preemptive and multimodal analgesia is often arranged in patients with orthopedic trauma when the patients are hospitalized. Meanwhile, preemptive and multimodal analgesia therapy has been proven to alleviate the patient’s pain sensation and accelerate recovery [23]. When the patient’s pain was controlled, the incidence of sleep disorders in this study was still highly prevalent. Therefore, orthopedic physicians should realize that merely controlling physical pain cannot significantly improve the quality of sleep of patients. These physicians also need to pay attention to the psychological problems of patients after trauma.

Poor sleep quality is independently associated with physical disability [6, 7]. At the same time, sleep disorders may be a core feature of PTSD [13]. Therefore, comprehensive physical and psychological management is essential for patients with acute orthopedic trauma. Patients with sleep disorders should be identified early and treated as soon as possible, which is of great significance for the accelerated recovery of patients.

Among the demographic indicators, we selected age, sex, marital status, and educational attainment to perform univariate linear regression analysis with PQSI scores. A multicenter cross-sectional cohort study of 4399 outpatients showed that the incidence of sleep disorders is different between men and women in China [24]. In this study, women had a higher incidence of sleep disorders than men. Sleep disorders were also highly correlated with marital status, and women who were divorced or widowed were more likely to suffer from sleep disorders [25]. Sleep disorders were associated with lower educational attainment in men [24]. Similar results were obtained in the present study. In univariate regression, female sex and low educational attainment were associated with poor sleep quality, but the marital status was not related. After eliminating confounding factors among multiple factors, only sex was independently related to sleep disorders. This suggests that sleep disorders in patients with acute orthopedic trauma are still unique compared to those in the overall population. The company of relatives and a high level of education may help stabilize the patient’s mood and sleep quality; however, our results showed that the amount of help these provided to patients was limited. Necessary psychotherapy is still critical for patients who suffer from sleep loss, especially in female patients.

In addition, we found that the ADL and the severity of trauma had a significant impact on the patient’s sleep quality in our study. Most orthopedic trauma patients (71.2%) were severely dependent even if they had suffered only mild injuries (81.8% of patients had mild injuries). Most orthopedic patients experience a certain degree of loss of limb function. Even if the injury is minor, they still cannot complete some normal daily activities, such as getting out of bed, combing their hair, and washing their face. No related literature has been found to prove that ADL in orthopedic trauma patients is directly related to sleep quality; however, there is literature to explain and illustrate that the decline in ADL may lead to depression and sleep disorders [26]. Stronger evidence is needed to prove the relationship between ADL and sleep disorders. At the same time, we observed that the severity of trauma was independently related to sleep quality. In the orthopedic ward of our trauma center, in addition to the patients in this city, we also accepted critically ill patients from other cities in Southwest China. Therefore, a sufficient number of patients with multiple, severe injuries were included in the study. These patients had higher ISS scores and worse sleep quality. In a previous study, the ISS scores of orthopedic trauma patients were positively correlated with somatic anxiety [27]. A higher degree of anxiety seriously affects the patient’s sleep quality, leading to sleep disorders. There is also literature showing that the severity of trauma is not related to sleep quality [14]. This result may be related to the low proportion of patients with multiple injuries included in this study. This requires prospective cohort studies to further confirm the relationship between trauma severity and sleep quality.

The biggest limitation of our study was its retrospective cross-sectional case analysis. All data were obtained from the nurses’ assessment of the patient’s admission. Sleep quality assessment also came from clinical nurses rather than professional investigators. This does not guarantee the consistency of the assessments. At the same time, the ISS score lacks precise numerical values, as it was only a grade evaluation, so the statistical validity was insufficient. Finally, we only collected data from a single level 1 trauma center. Prospective comparative studies are needed to further clarify the impact of relevant risk factors on patients' sleep quality to establish clinical prevention methods.

## Conclusions

Sleep disorders in patients with acute orthopedic trauma are very common and severe. A patient’s sex, ability to perform daily living activities, and severity of trauma significantly affect sleep quality. However, age, marital status, and educational attainment have a limited influence on sleep quality. Being female, poor ability of daily living, and severe physical trauma should be accepted as independent risk factors for sleep disorders in patients with acute orthopedic trauma.

## Data Availability

Datasets are available from the corresponding author on reasonable request.

## Abbreviations

PSQI: Pittsburgh sleep quality index
VAS: Visual analog scale
ISS: Injury Severity Score
BI: Barthel Index
ADL: Activities of daily living
